# Implementing drinking water feed additive strategies in post-weaning piglets, antibiotic reduction and performance impacts: case study

**DOI:** 10.1186/s40813-016-0043-0

**Published:** 2016-10-16

**Authors:** Juan Antonio Mesonero Escuredo, Yvonne van der Horst, John Carr, Dominiek Maes

**Affiliations:** 1 Resident European College of Porcine Health Management, University of Ghent, Ghent, Belgium; 2 Nutreco BV, Trouw Nutrition Selko, Jellinghausstraat 24, Tilburg, P.O. Box 4217, 5004 JE Tilburg, The Netherlands; 3 Howells Veterinary Services, Easingwold, UK; 4 Faculty of Veterinary Medicine, Department of Reproduction, Obstetrics and Herd Health, Unit Porcine Health Management, Ghent University, Salisburylaan 133, B-9820 Merelbeke, Belgium

**Keywords:** Piglets, Organic acids, Additives, Drinking water, Post-weaning period, Nursery, Performance, Antibiotic reduction

## Abstract

**Background:**

Piglets at weaning suffer many stressors such as sudden change of feed, change in group composition and the end of lactogenic immunity. These stressors may cause poor growth performance. There is a need for alternatives to support piglets during the weaning period. Organic acids are known to have a positive effect on performance through reducing the pH and their antimicrobial action.

**Context & purpose:**

The purpose was to study the effect of the inclusion of a free and buffered organic acid blend in drinking water on performance of weaned pigs.

Four-hundred and twenty pigs in a conventional herd were allocated after weaning to one of three treatments and monitored during 4 weeks: group (1) Full medication, group (2) organic acid blend + full medication, group (3) organic acid blend + reduced medication. Average daily gain, feed intake and water consumption was recorded at group level.

**Results:**

During the overall study period live weight and average daily gain of the piglets was significantly higher (*P* <0.001) for treatment (3) compared to (1) and (2) (Table 1). Live weight was significantly higher for treatment (3) compared to (1) from week 2 of the study (Fig. 1). No significant differences were found for average daily feed intake. FCR for treatment (3) improved by 1.0 compared to treatment (1) in week 1 (*P* <0.05), while in week 2 and 3 no significant differences were found (Table 2). Overall, FCR was with 0.3 difference significantly lower (*P* = 0.001) for treatment (3) than for (1) and (2) (Table 1). Pigs receiving organic acids in drinking water had significantly (*P* <0.05) higher water consumption than group (1) in weeks 3 and 4 (Table 2).

**Conclusion & potential implications:**

The use of a blend of free and buffered organic acids together with a reduced medication program improves growth performance during the first month after weaning compared to a control with full medication and a combination between organic acids and full medication. This implies that organic acids could be used as a valid alternative for antibiotic reduction in post-weaning pigs. The treatment also increased the drinking water intake.

**Electronic supplementary material:**

The online version of this article (doi:10.1186/s40813-016-0043-0) contains supplementary material, which is available to authorized users.

## Background

Weaned piglets are exposed to many stressors, such as abrupt change of feed, change in group composition and the end of lactogenic immunity [11, 20]. As a consequence, malabsorption problems might occur resulting in poor growth performance and increased mortality. Antimicrobials have been used for more than 50 years to increase growth performance and to prevent piglet diseases after weaning [7, 10]. The increasing resistance of bacteria against antibiotics has resulted in a ban on antibiotic growth promoters in feed in the European Union in 2006 (EU Regulation 1831/2003) and several programs have been developed worldwide until today to reduce the clinical application of antibiotics. This has urged the need for alternatives which have antibacterial and growth promoting effects, without inducing resistance.

Organic acids have been shown as viable growth promoters in swine diets [16, 17, 21]. The antimicrobial effect of organic acids in pigs is suggested to be achieved in several ways. Firstly, uptake of pathogenic bacteria can be reduced by lowering the pH of feed and water. Chaveerach et al. [3] demonstrated that survival of *Campylobacter jejuni* in a feed/water mixture declined to below detection limits within one hour of incubation when formic, propionic or acetic acid was added until pH 4.0 was reached. Organic acids are also suggested to reduce pH-value in the stomach of recently weaned pigs [9]. A lower gastric pH results in a stronger biological barrier function of the stomach against bacterial transfer to the intestine and it could result in increased activity of proteolytic enzymes [9]. Furthermore, it has also been shown that *Lactobacilli* are better resistant to acidic conditions in the stomach, which possibly inhibits the colonization and proliferation of *E.coli* [5] through mutual exclusion. Secondly, un-dissociated forms of organic acids can penetrate the wall of the bacterial cell and once inside it will dissociate into anions and protons [4]. This will disrupt internal metabolism of bacteria and inhibit proliferation. In the review of Canibe et al. [1] several sources describe that the application of organic acidifiers in the diet can reduce the number of *Coliform* bacteria along the intestinal tract of piglets (testing method not specified in the review). Bacteria like *Lactobacilli* and *Bifidobacterium spp*. tolerate a larger change in internal pH and the bacteria will not suffer from the acid molecules that have entered the bacterial cell.

Organic acids can be applied to the pigs through water or through feed. Water intake of pigs is approximately 2–3 times as much as the intake of feed [13]. Therefore, application of organic acids through water will result in a higher level of acid intake and consequently a higher amount of acid reaching the intestinal tract of the animal when compared to application through feed when the acid is included at an equal concentration in both feed and water. Furthermore, supplying acids via water provides much more flexibility, since the dosage can be changed per day. This enables the farmer to adapt the dosage of acids to the needs of the animals.

In this study we investigated the effect of organic acids on the possibility to reduce medication without reducing performance. In most of the previous studies organic acids were tested alone instead of in combination and no comparison was made with application of medication.

The objective of the present study was to investigate the effect of application of a blend of free and buffered organic acids in the drinking water (Additional file 1) on performance of weaned piglets, both in the presence and absence of antibiotic medication used to control enteric disease.

## Materials and methods

### Study farm and study animals

The study was performed in the nursery of a commercial research facility at Westbrook, Qld (Australia). One-hundred and forty (140) weaned pigs (21 days Genetic hybrid slaughter line, Large with Landrace and Duroc mix from PIC) entered the facility each week with 3 weeks of entries being used, resulting in four-hundred and twenty (420) pigs in total in this study (Additional file 2).

The herd was positive for *Mycoplasma hyopneumoniae* and *Actinobacillus Pleuropneumoniae* (Serovar 15). The pigs were vaccinated against *M. hyopneumoniae* (1 mL/pig M+PAC, Merck Animal Health) at 3 and 9 weeks of age. Vaccination against *A. Pleuropneumoniae* (2 mL/pig Porcilis APPvac, Merck Animal Health) was applied at 9 and 12 weeks of age and at 12 and 14 weeks of age the pigs were vaccinated against GnRF (2 mL/pig Improvac, Zoetis). Clinical problems due to *Haemophilus parasuis* were not present on the sow farm, only at the research facility. The most recent test on small intestine swabs for *E.coli* that was done on the research facility was O139:K88 positive.

Removal of pigs from the experimental groups was based on body condition and assessment of clinical state (i.e. alert vs. depressed/listless).

Penning was open galvanized paneling with fully-slatted plastic floor tiles. All pens in the nursery were of identical configuration (1 m × 2.8 m).). A total of 30 pens were included in the study (420 piglets), ten pens per treatment (140 piglets), with 14 piglets per pen (one replica each pen). The climate control in the facility was achieved through natural ventilation by manually controlled side curtains. Radiant bar heaters were present in each pen with a temperature probe and controller for the block of ten pens. Water was supplied ad libitum via two nipple drinkers per pen. Feed was offered in pellet form to each individual pen via a round multi-space adjustable plastic transit feeder. Diets were offered ad libitum throughout the experimental period.

Before the pigs entered the facility, the water lines were flushed with the blend of organic acids during 1 week at a dosage of 2 L per 1000 L of water in order to start with a clean drinking water system.

### Experimental design

Upon entry, pigs were sexed and graded into large, medium and small pigs and assigned a pen (*n* = 14). Pigs were weighed at pen-level (average entry weight 6.1 ± 0.14 kg) and allocated to treatment using a randomized block design with sex, weight and entry date as blocking factors. All piglets in the study received feed medication with *Amoxycillin trihydrate* dosed at 400 ppm during the duration of the study to prevent problems with *H. parasuis* infections.

The below three treatments were applied:

1. NEO- *Neomycin sulfate* 300 ppm in feed
2. OA + NEO - OA blend at 2 L/1,000 L of water + *Neomycin sulfate* 300 ppm in feed
3. OA - OA blend at 2 L/1,000 L of drinking water

An individual block within the weaned facility consisted of ten pens per treatment with 14 pigs per pen. The block refers to the week of entry of the pigs, therefore we apply all treatments in all weeks as a way of accounting for this block. As we are applying, in this experiment, three treatments to ten pens each weeks, it’s not always going to be possible to complete balance the treatments for weights. Whilst we are seeing main effects from treatments across the experiment, there is also a consistent block effect.

**Fig. 1:**
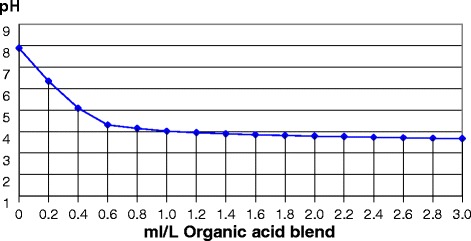
Water titration results of the drinking water with a blend of formic acid, acetic acid and ammonium formate of the weaned pigs prior to onset of this study

The organic acid blend consisted^1^ of formic acid, acetic acid and ammonium formate and was delivered at the rate of 2 L /1,000 L of drinking water. This dosage was determined based on a water titration (Fig. 1). The water consumption was measured via individual water meters in each pen. Medications used in all treatments were administered in-feed under veterinary advice. *Neomycin sulphate* (600 mg/g – 0.5 kg/t (300 ppm) in feed) was applied to prevent problems with post-weaning *E.coli* infections. Samplings occurred as a response to a period of increased morbidity or mortality on farm. Samples were obtained from euthanized pigs showing signs of significant ill-thrift. Two swabs from intestines showing gross pathology of *E.coli* infection were cultured and one lung swab was cultured from a set of lungs showing signs of congestion. Sensitivity test indicates *E. coli* was sensitive to *Neomycin* and *Ceftiofur*, but resistant to other antibiotics. No lung pathogens were cultured. Hemolytic *E.coli* was cultured from small intestine swabs, it was resistant to *Amoxycillin*, *Apramycin*, *Florfenicol, Lincomycin and streptomycin combination*, *Trivetrin*. The study lasted for 4 weeks.

### Parameters of comparison

#### Average daily weight gain

Pens were weighed weekly and the average of the number of piglets in the pen was calculated, averaged over 7 days. ADG was calculated every 7 days, and was on the basis of average pig live weight of each pen at the start and the end of week.

#### Feed conversion ratio

Weekly feed disappearance was calculated from feed deliveries and weighed refusal on the final day of the week. ADFI was calculated by dividing the total feed consumption in a week by the number of pig days. FCR is calculated by dividing ADFI by ADG.

#### Morbidity

Morbidity was determined by an extended period (3 days) of listlessness based on posture and behavior.

### Data and statistical analyses

The null hypothesis was that the inclusion of OA to the drinking water could not enhance growth performance (i.e. body weight and feed conversion ratio) compared to treatment NEO nor reduce the reliance on medication.

Data were analyzed via an unbalanced ANOVA with week of entry as a blocking factor and entry weight as a covariate, pairwise differences between treatments were determined by LSD (*P* <0.05). Removals were tested for significance via Chi-square analysis (*P* <0.05). A sample size analysis (one-sided t-test; significance level 5 %; power 80 %) was ran in order to test the statistical power of the study and showed that the study had adequate sample size for the differences found.

Mortality analysis was done through Pearson’s chi-square of sample distribution, comparing total deaths/removals and live pigs at the end of experimental period (GenStat 15^th^ edition (VSN International, Hemel Hempstead, UK)).

## Results

Pigs receiving the OA treatment were significantly heavier at the end of the experimental treatment than the NEO and OA + NEO treatments, with a difference of 1 Kg between the OA and NEO group (Table 1). The pigs from the OA treatment tended to grow faster than the NEO and OA + NEO treatments during each week, but the difference was only significant during week 4 (*P* <0.001) (Table 2). Over the total duration of the study average daily gain was higher for the OA treatment compared to the NEO and OA + NEO treatment (*P* <0.001) (Table 1). There was no significant difference in feed intake between treatments over the complete duration of the trial or per week (Tables 1 & 2). Over the total duration of the study, feed conversion ratio was significantly better for the OA treatment compared to the NEO and OA + NEO treatment (Table 1). Feed conversion ratio for the OA treatment improved by 1.0 compared to the NEO and OA + NEO treatment in week 1 (*P* <0.05), while in week 2 and 3 no significant differences were found. In the fourth week of the study feed conversion ratio was improved by 0.3 in the OA treatment compared to the NEO and OA + NEO group (*P* = 0.001) (Table 2). In the results per week live weight in week 4, feed conversion ratio in week 1 and average daily gain in week 4 showed significant interactions with blocking factors (sex, weight and entry date). The cumulative data showed a significant interaction with blocking factors for FCR after week 1 and 2 and for average daily gain after week 4.

**Table 1:** Cumulative average daily gain (ADG), average daily feed intake (ADFI), feed conversion ratio (FCR) per week of 140 weaned piglets (21 days) supplied with or without an organic acid (OA) blend in the drinking water and with full or reduced medication program in the feed

	Control	OA + NEO	OA	SED	*Trtmt*	*Block*	*T x B*
Live weight	12.5^a^	12.8^a^	13.5^b^	0.24	<0.001	<0.001	0.008
ADG	0.228^a^	0.238^a^	0.265^b^	0.009	<0.001	0.008	0.008
ADFI	0.40	0.40	0.39	0.024	0.738	0.497	0.229
FCR	1.76^a^	1.70^a^	1.46^b^	0.073	0.001	0.520	0.113
Mortality	0	3	3				
Morbidity	3	4	9				
Total Mortality + Morbidity	3^a^	7^ab^	12^b^				

^a,b^Means in a row with different superscripts differ significantly (*P* <0.05); Removals were tested for significance via Chi-square analysis, χ2 (2, *N* = 140) = 5.95, *P* = 0.051

1. Control - Neomycin sulfate 300 ppm in feed

2. OA + NEO - OA blend at 2 L/1,000 L of water + Neomycin sulfate 300 ppm in feed

3. OA - OA blend at 2 L/1,000 L of water

**Table 2:** Data per week with or without additives, with or without reducing antibiotics; Live weight, average daily gain (ADG), average daily feed intake (ADFI), feed conversion ratio (FCR) per week of 140 weaned piglets (21 days) supplied with or without an organic acid (OA) blend in the drinking water and with full or reduced medication program in the feed

		Control	OA + NEO	OA	SED	*Trtmt*	*Block*	*T x B*
Live weight	Entry	6.2	6.1	6.0	0.37	0.893	0.266	0.809
	Week 1	6.5	6.5	6.7	0.08	0.074	<0.001	0.294
	Week 2	8.0^a^	8.1^ab^	8.3^b^	0.12	0.026	<0.001	0.086
	Week 3	9.8^a^	10.0^a^	10.3^b^	0.22	0.043	<0.001	0.188
	Week 4	12.5^a^	12.8^a^	13.5^b^	0.24	<0.001	<0.001	0.008
Week 1	ADG	0.052	0.056	0.076	0.012	0.081	0.073	0.310
	ADFI	0.16	0.16	0.17	0.014	0.458	0.200	0.747
	FCR	3.51^a^	3.04^ab^	2.48^b^	0.398	0.043	0.073	0.032
Week 2	ADG	0.218	0.220	0.232	0.010	0.182	0.003	0.108
	ADFI	0.33	0.33	0.32	0.020	0.941	0.180	0.513
	FCR	1.53	1.49	1.40	0.079	0.151	<0.001	0.296
Week 3	ADG	0.266	0.282	0.298	0.018	0.145	<0.001	0.399
	ADFI	0.48	0.48	0.45	0.032	0.467	0.780	0.144
	FCR	1.84	1.73	1.54	0.115	0.059	<0.001	0.508
Week 4	ADG	0.403^a^	0.419^a^	0.478^b^	0.018	<0.001	0.003	0.015
	ADFI	0.64	0.67	0.63	0.041	0.475	0.256	0.145
	FCR	1.62^a^	1.62^a^	1.31^b^	0.081	0.001	0.022	0.099

1. Control - Neomycin sulfate 300 ppm in feed

2. OA + NEO - OA blend at 2 L/1,000 L of water + Neomycin sulfate 300 ppm in feed

3. OA - OA blend at 2 L/1,000 L of water

^a,b^ Means in a row with different superscripts differ significantly (*P*<0.05); ADG, average daily gain; ADFI, average daily feed intake; FCR, feed conversion ratio; SED, standard error of difference of the means

Pigs receiving the OA and the OA + NEO treatment in water had significantly higher water consumption than the NEO in weeks 3 and 4 (Table 3).

**Table 3:** Water consumption L/pen/week; Water usage of 140 weaned piglets (21 days) supplied with or without an organic acid (OA) blend in the drinking water and with full or reduced medication program in the feed (L/pen/week)

		Control	Control + OAs	OAs + red.	SED	*Treatment*	*Block*	*T x B*
Water consumption	Week 1	7.5	8.4	9.2	2.05	0.628	0.005	0.967
	Week 2	8.7	17.2	13.9	3.61	0.079	0.021	0.639
	Week 3	11.4^a^	24.5^b^	23.8^b^	3.67	0.004	0.691	0.416
	Week 4	13.9^a^	31.6^b^	28.4^b^	5.65	0.014	0.880	0.824

**Table 4:**
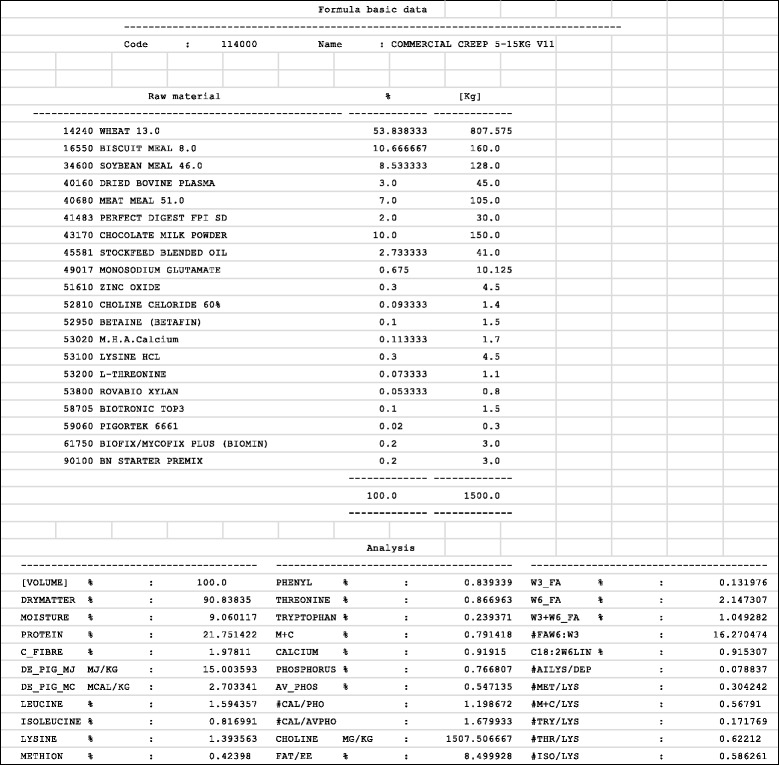
Basic feed formula data

Mortality and morbidity rates were highest in the group receiving the OA treatment (*P* <0.05). No difference was found between the NEO and OA + NEO treatment (Table 1).

## Discussion

In this study it was shown that application of organic acids in drinking water of piglets over a period of 4 weeks improved performance when levels of medication are reduced. Significant improvements are found in final weight, average daily gain and feed conversion ratio. An increase in mortality rates was found in the group with organic acids with reduced medication.

### Weight and average daily gain

The increasing effects of organic acids on live weight and average daily gain are commonly seen in studies with organic acids in the diet. In general, literature data show a similar effect of dietary organic acids in young piglets. Kirchgessner et al. 1992 and Kim et al. 2004 found that supplementation of formic acid at levels up to 1.2 % in diets of weaning piglets of 6 to 12 kg of body weight improved growth rate to a maximum of 31 %. At concentrations higher than 1.2 % the effect of formic acid on growth was less efficient. Lawlor et al. [12] demonstrated that increases in ADG occurred in response to inclusion of 0.2 % fumaric acid in the diet mainly in the initial 2 weeks post weaning, and confirmed that these effects have been noted previously by Radecki et al. [18] and Giesting et al. [6]. It has been suggested that the improvement in growth is the result of a reduced pH in the stomach induced by the organic acids. As a result, there is a better barrier function of the stomach against harmful bacteria to enter into the small intestine and a more efficient protein digestion [9, 22]. In a meta-analysis by Partanen and Mroz [17], it was shown that organic acids generally improved growth performance of piglets, but the effects vary greatly. Reasons for the varying results relate to dosage and type of acids used, composition of basal diet, age of piglets, and existing levels of performance [19]. The latter might also explain the interaction found between treatment and block for live weight and average daily gain in weekly and cumulative data.

### Feed intake

Feed intake was not affected as a result of the OA and OA + NEO treatment in the current study. The organic acids were applied in the drinking water instead of in the feed, therefore less effects on feed intake could be expected compared to when the acids would have been applied in the feed. Several former studies report on the effect of acids in the feed on feed intake. Lawlor et al. [12] showed that in two out of three experiments feed intake was not affected by feeding diets containing fumaric acid or calcium formate. Increases in feed intake and average daily gain were mainly found in the initial 2 weeks after weaning [17]. Literature data indicate that a higher feed intake might be related to better diet palatability [17], but the extent of the effect is dependent on the type of organic acid added. The sources used in the review of Partanen and Mroz [17] indicate that in general formic acids and formates have a positive effect, fumaric no effect and citric acid had a negative effect.

### Feed conversion ratio

Overland et al. [15] found that dietary addition of formic, benzoic and sorbic acid in the diet significantly improved FCR compared with a NEO group without dietary organic acids. Canibe (2005) demonstrated that pigs in the growth phase of 27 to 99 kg BW had a better G:F when supplemented with formic acid than those fed a diet without formic acid (392 g/kg vs. 351 g/kg respectively, *P* = 0.02). The more efficient utilization of feed is also expected to be the result of the stronger pH reduction and consequent increase in level of active protein digestion enzymes in the stomach.

### Water consumption

In the present study, water consumption of the piglets was higher for the OA and OA + NEO, with significant differences found in week 3 and 4 of the study. Houben et al. [8] found that water consumption of pigs supplied with water with a low pH-value (3.4–3.8) was clearly higher compared to pigs supplied with non-acidified water (pH value 6.7–7.8). It is not clear from the present study if the higher intake is the result of a better palatability because of the acids, since no opportunity was given for choosing between acidified and non-acidified water and the sodium content of water differed between treatments.

#### Mortality and medication

Mortality was significantly higher for the OA with reduced medication treatment compared to the full medication treatment and the control. Gross post-mortems on farm were consistent with *H. parasuis* in two deaths in NEO + OA and three deaths in Selko-pH treatment. 1 death within the NEO + OA treatment was not able to be determined through post-mortem. This shows that the mortality was due to causes that cannot be controlled by organic acids.

### Limitations of the study

In the current study only one piglet herd was included, which may affect the results compared to when multiple herds from different origin are used since the animals can have a different status at the start of the trial. Because there was no control treatment without antibiotics, there is no data about the performance of pigs without any treatment.

The first 4 weeks are the most important in the growth and development of piglets, therefore only these weeks were included in the trial. Therefore, there are no data available on the weeks after that.

No data is available on the microbiota composition and pathogenic bacteria levels in the feces of the pigs, therefore no conclusion can be given on the effect of the OA or OA + NEO treatments on the microbial balance of the pigs. In the literature several sources indicate a positive effect of organic acids on the microbiota of pigs [2, 14, 15, 23].

## Conclusions

The use of a blend of free and buffered organic acids improved live weight, ADG and FCR compared to a combination of the blend of acids with *Neomycin* against post-weaning *E. coli* infections during the first month after weaning. This implies that OA could be used as a valid alternative for antibiotic medication in pigs post-weaning. The treatment also increased the drinking water intake. Further research should be conducted including more herds with different management practices to confirm the present results, and to elucidate the mechanisms that are responsible for the beneficial effects.

## Additional files

Additional file 1: Water analysis. (PDF 330 kb)

Additional file 2: Facility Pictures. (ZIP 2811 kb)

## Abbreviations

ADFI: Average daily intake
ADG: Average daily gain
d: Days
FCR: Feed conversion rate
MCFA: Medium chain fatty acids
med.: Medication
OAs: Blend of free and buffered organic acids (Formic acid, acetic acid and ammonium formate)
red.: Reduced
SCFA: Short chain fatty acids
SED: Standard error of difference of the means
T x B: Treatment by block
Trtmt: Treatment

## Footnotes

1 35 % formic acid, 9 % acetic acid, 25 % ammonium formate, <1 % copper sulphate.
