# Supplementary material for: Implementing drinking water feed additive strategies in post-weaning piglets, antibiotic reduction and performance impacts: case study
Source: Porcine Health Manag. 2016 Oct 16;2:25. doi: 10.1186/s40813-016-0043-0 (PMC5382475; doi:10.1186/s40813-016-0043-0)
Supplement: Supplementary file 1 — Water analysis. (PDF 330 kb) [file 40813_2016_43_MOESM1_ESM.pdf]

Issued: 13/05/14

BEING WATERWISE
TOOWOOMBA REGIONAL WATER
ABN 99 788 305 360
LABORATORY SERVICES
Shuttlewood Court Mt Kynoch Toowoomba Qld 4350
PO Box 3021
Toowoomba Village Fair Qld 4350
Email: labservices@toowoombaRC.qld.gov.au
T: 07 4688 6270 F: 07 4688 6259

TEST REPORT

BATCH NO: 14/1510
RECEIVED: 7/05/14
APPROVED: 13/05/14

CLIENT:
CHM Alliance Pty Ltd
210 Bunkers Hill School Road
Westbrook QLD 4350
ATTENTION: Andres Corso

ORDER NO:

REPORT NO: 070514-1510-1

METHOD	Client Reference:<br>Laboratory Reference:<br>Sample Date:<br>Sample Time:<br>ANALYSIS	UNITS	LOR	Bore Water<br>14/1510/1<br>7/05/14<br>9:30am
QP-KYN-001	pH	UNITS		8.9
QP-KYN-002	Conductivity	uS/cm	1	422
QP-KYN-017	Total Hardness	mg/L $CaCO_3$	1	6.4
QP-KYN-015	Total Alkalinity	mg/L $CaCO_3$	2	120
QP-KYN-090	Molybdate Reactive Silica	mg/L	1.0	22.4
QP-KYN-014	Total Iron	mg/L	0.01	0.04
QP-KYN-014	Total Manganese	mg/L	0.01	<0.01
QP-KYN-016	Calcium	mg/L	1	2.6
Derived*	Magnesium	mg/L	2	<2.0
QP-KYN-014	Sodium	mg/L	0.5	85.0
QP-KYN-014	Potassium	mg/L	0.1	0.4
QP-KYN-058	Sulphate	mg/L SO4	1	7
QP-KYN-058	Chloride	mg/L	1	59
QP-KYN-058	Nitrate	mg/L NO3	1	<1.0
QP-KYN-022	Phosphate	mg/L PO_4	0.02	0.06
QP-LSB-A013	Temporary Hardness	mg/L $CaCO_3$	1	6.4
QP-LSB-A013	Bicarbonate Alkalinity	mg/L $CaCO_3$	1	104
QP-LSB-A013	Carbonate Alkalinity	mg/L $CaCO_3$	2	16
QP-LSB-A013	Hydroxide Alkalinity	mg/L $CaCO_3$	2	<2
QP-LSB-A013	Free Carbon Dioxide	mg/L	0.1	0.3
QP-LSB-A013	Total Dissolved Ions	mg/L	1	290
QP-LSB-A013	Total Dissolved Solids	mg/L	1	248
QP-LSB-A013	Figure of Merit		0.1	<0.1
QP-LSB-A013	Saturation Index			-0.13
QP-LSB-A013	Residual Alkalinity	meq/L $CaCO_3$		2.0
QP-LSB-A013	Sodium Adsorption Ratio		0.1	14.5

File Reference: S-002289 (External)
LOR = Limit of Reporting
QP-LSB-A013 - Derived value.
Results apply to sample(s) as received at laboratory.

This document may not be reproduced, except in full.

BEING WATERWISE
TOOWOOMBA REGIONAL WATER
ABN 99 788 305 360
LABORATORY SERVICES
Shuttlewood Court Mt Kynoch Toowoomba Qld 4350
PO Box 3021
Toowoomba Village Fair Qld 4350
Email: labservices@toowoombaRC.qld.gov.au
T: 07 4688 6270 F: 07 4688 6290

TEST REPORT

Issued: 13/05/14

BATCH NO: 14/1510
RECEIVED: 7/05/14
APPROVED: 13/05/14

CLIENT:

CHM Alliance Pty Ltd
210 Bunkers Hill School Road
Westbrook QLD 4350
ATTENTION: Andres Corso

ORDER NO:

REPORT NO: 070514-1510-1

Comments

All chemical parameters tested comply with the NHMRC Drinking Water Guidelines

J.L. MILLS
Principal Scientist Laboratory Services

This document may not be reproduced, except in full.

File Reference: S-002289 (External)
LOR = Limit of Reporting
QP-LSB-A013 - Derived value.
Results apply to sample(s) as received at laboratory.
