# Supplementary figures and images for: Implementing drinking water feed additive strategies in post-weaning piglets, antibiotic reduction and performance impacts: case study
Source: Porcine Health Manag. 2016 Oct 16;2:25. doi: 10.1186/s40813-016-0043-0 (PMC5382475; doi:10.1186/s40813-016-0043-0)

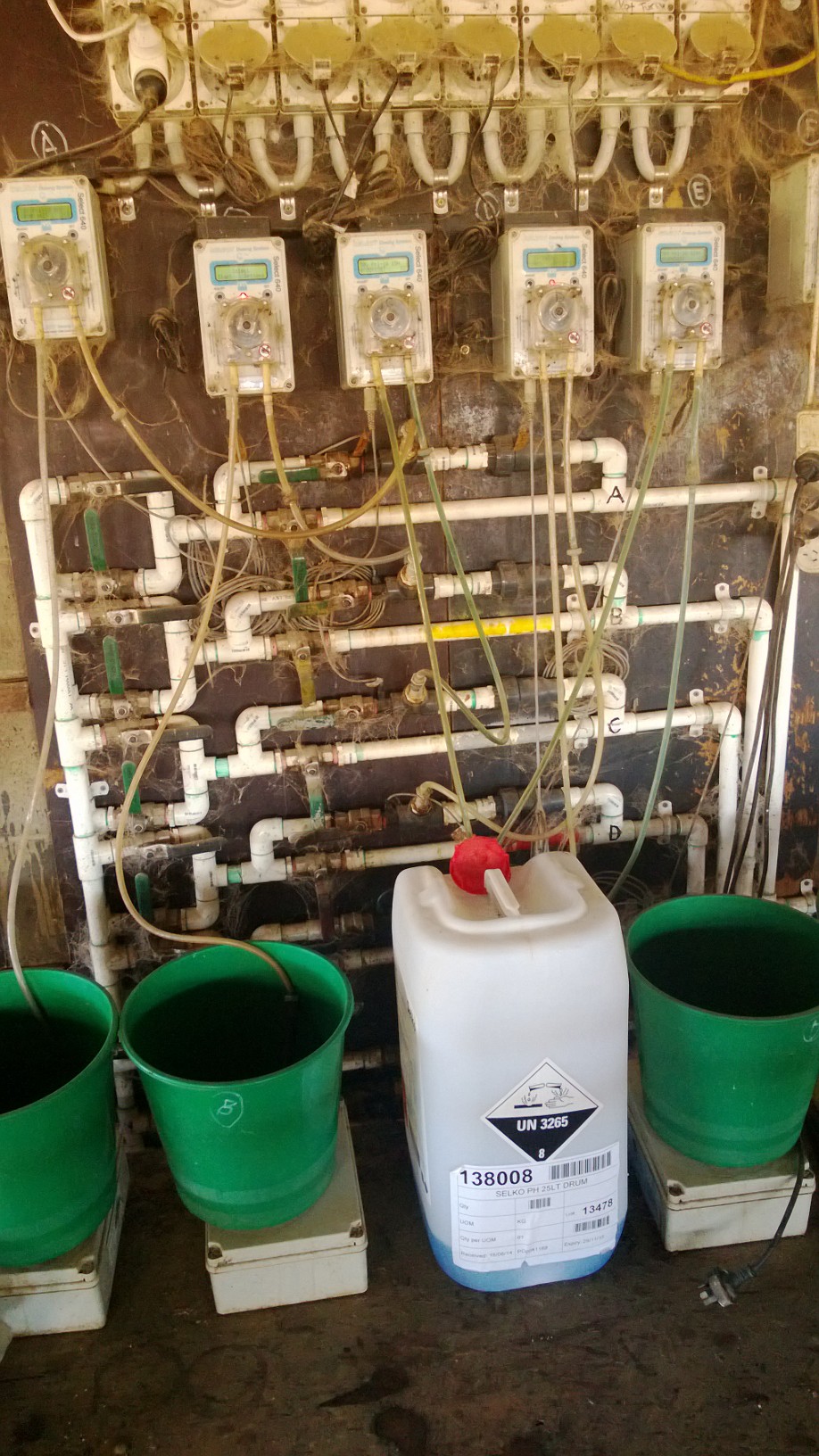

Supplement: Supplementary file 2 — Facility Pictures. (ZIP 2811 kb) [file 40813_2016_43_MOESM2_ESM.zip › Facility Pictures/Doser Setup.jpg]

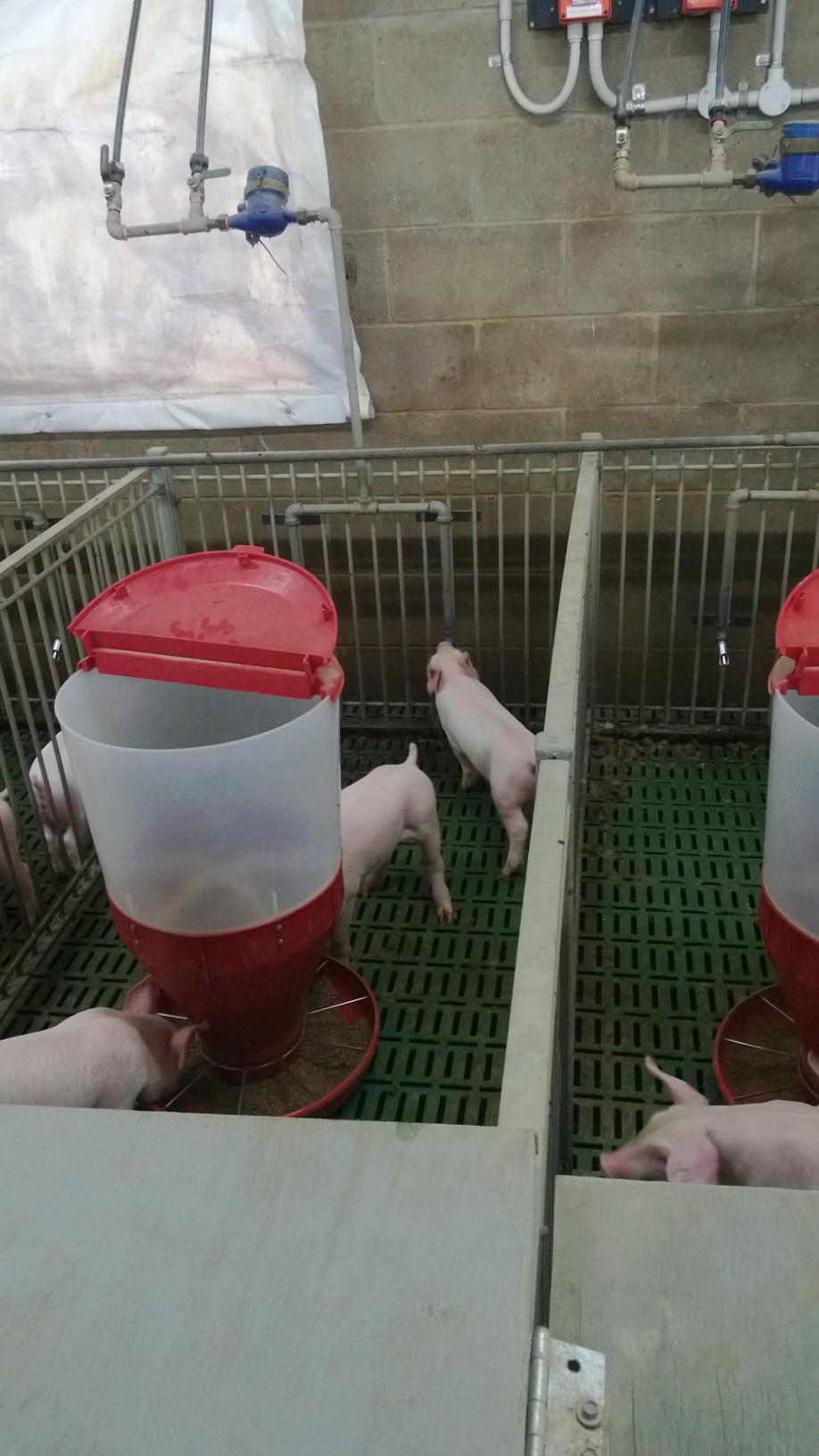

Supplement: Supplementary file 2 — Facility Pictures. (ZIP 2811 kb) [file 40813_2016_43_MOESM2_ESM.zip › Facility Pictures/Drinkers.jpg]

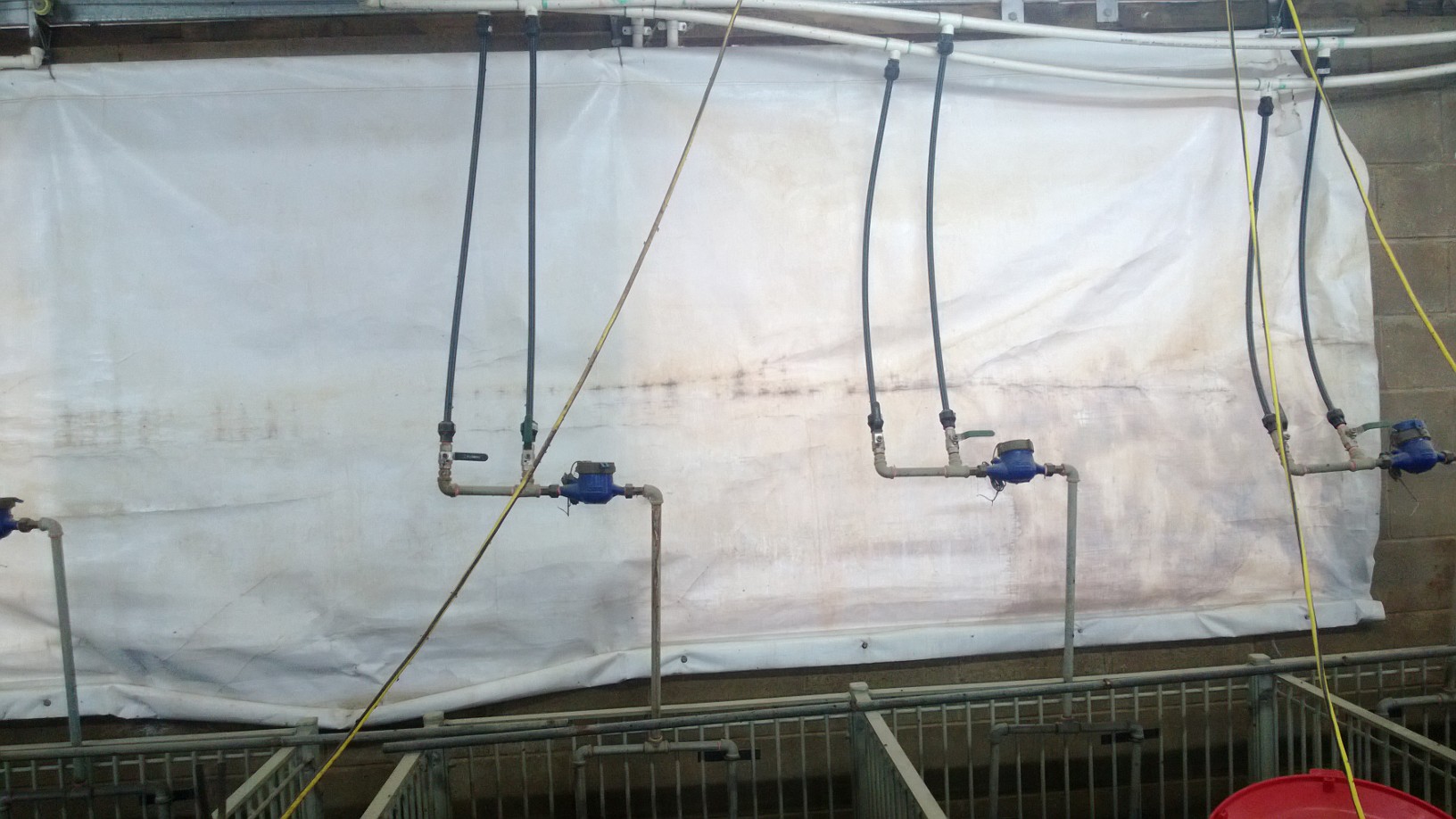

Supplement: Supplementary file 2 — Facility Pictures. (ZIP 2811 kb) [file 40813_2016_43_MOESM2_ESM.zip › Facility Pictures/Drinking water taps.jpg]

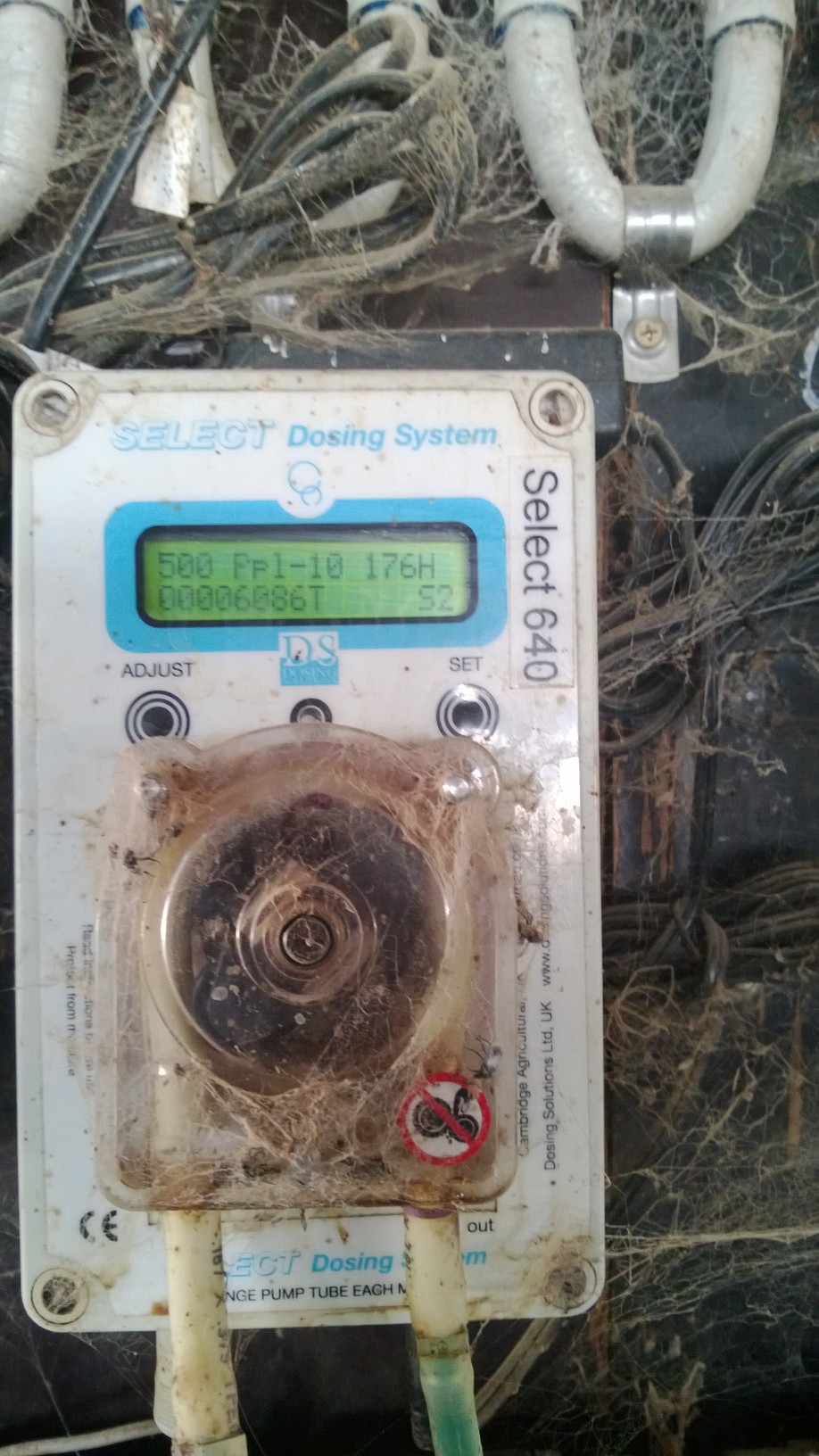

Supplement: Supplementary file 2 — Facility Pictures. (ZIP 2811 kb) [file 40813_2016_43_MOESM2_ESM.zip › Facility Pictures/Selko pH doser.jpg]

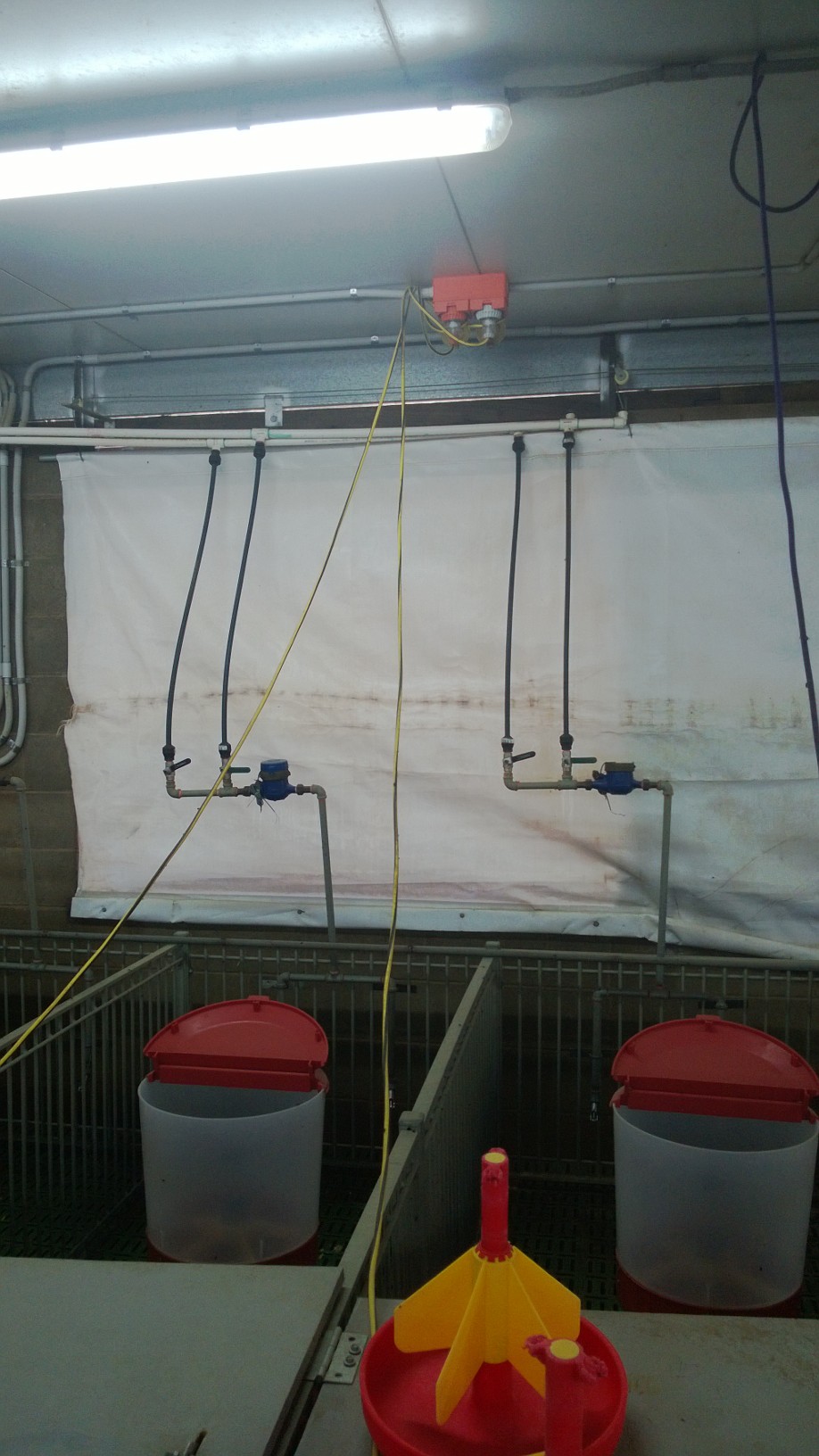

Supplement: Supplementary file 2 — Facility Pictures. (ZIP 2811 kb) [file 40813_2016_43_MOESM2_ESM.zip › Facility Pictures/Trial 1.jpg]

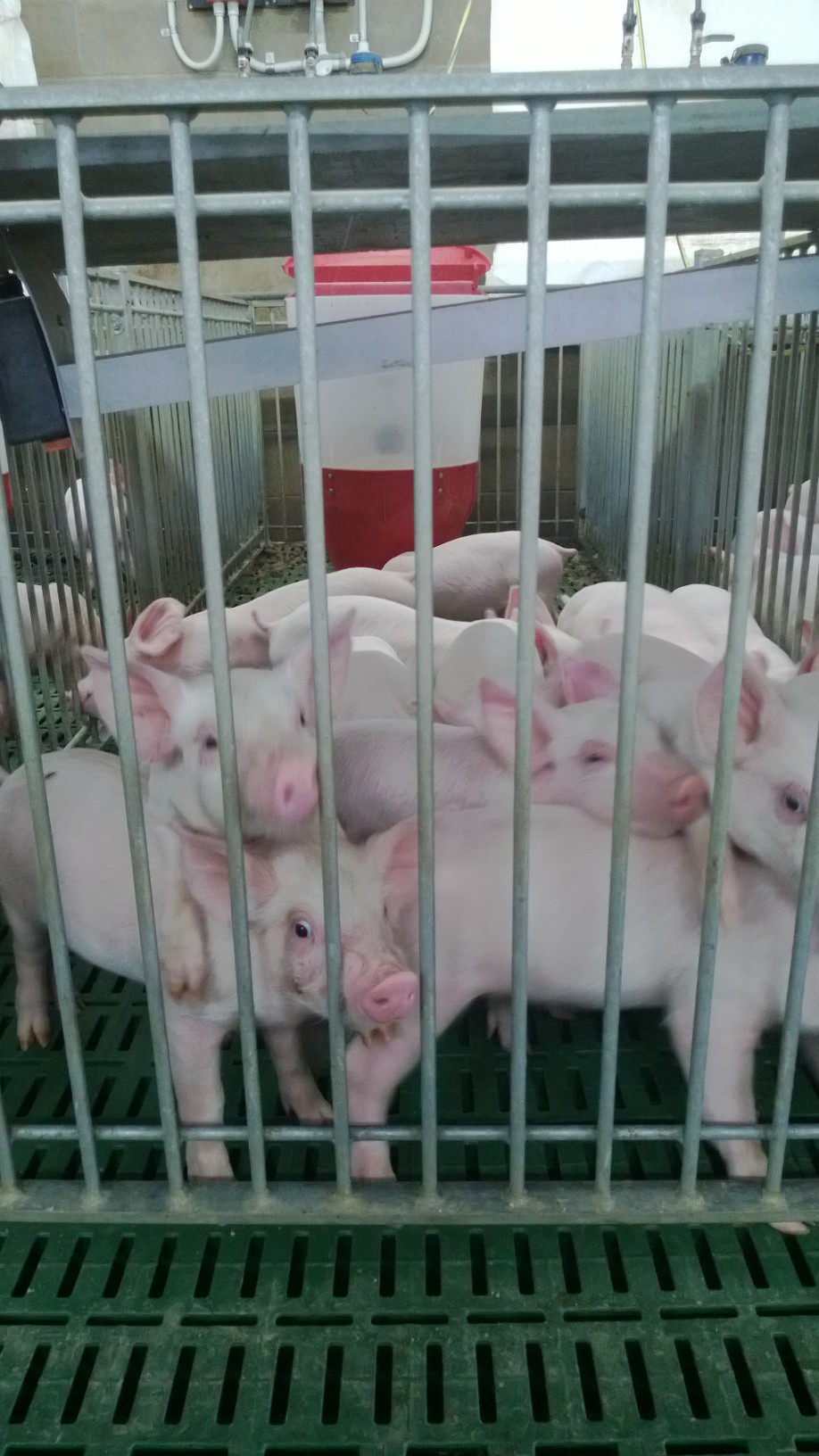

Supplement: Supplementary file 2 — Facility Pictures. (ZIP 2811 kb) [file 40813_2016_43_MOESM2_ESM.zip › Facility Pictures/Trial 2.jpg]

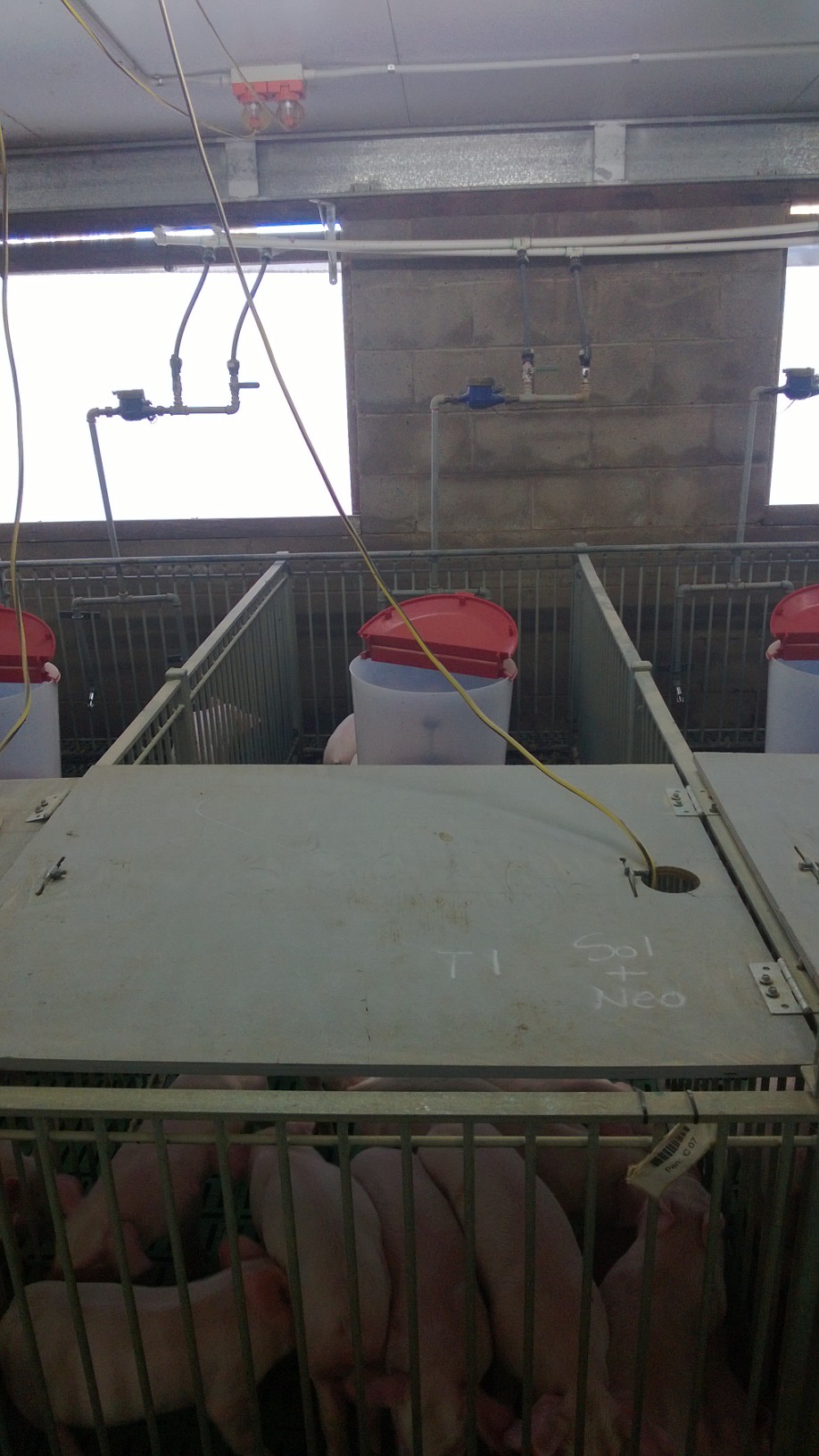

Supplement: Supplementary file 2 — Facility Pictures. (ZIP 2811 kb) [file 40813_2016_43_MOESM2_ESM.zip › Facility Pictures/Trial 3.jpg]

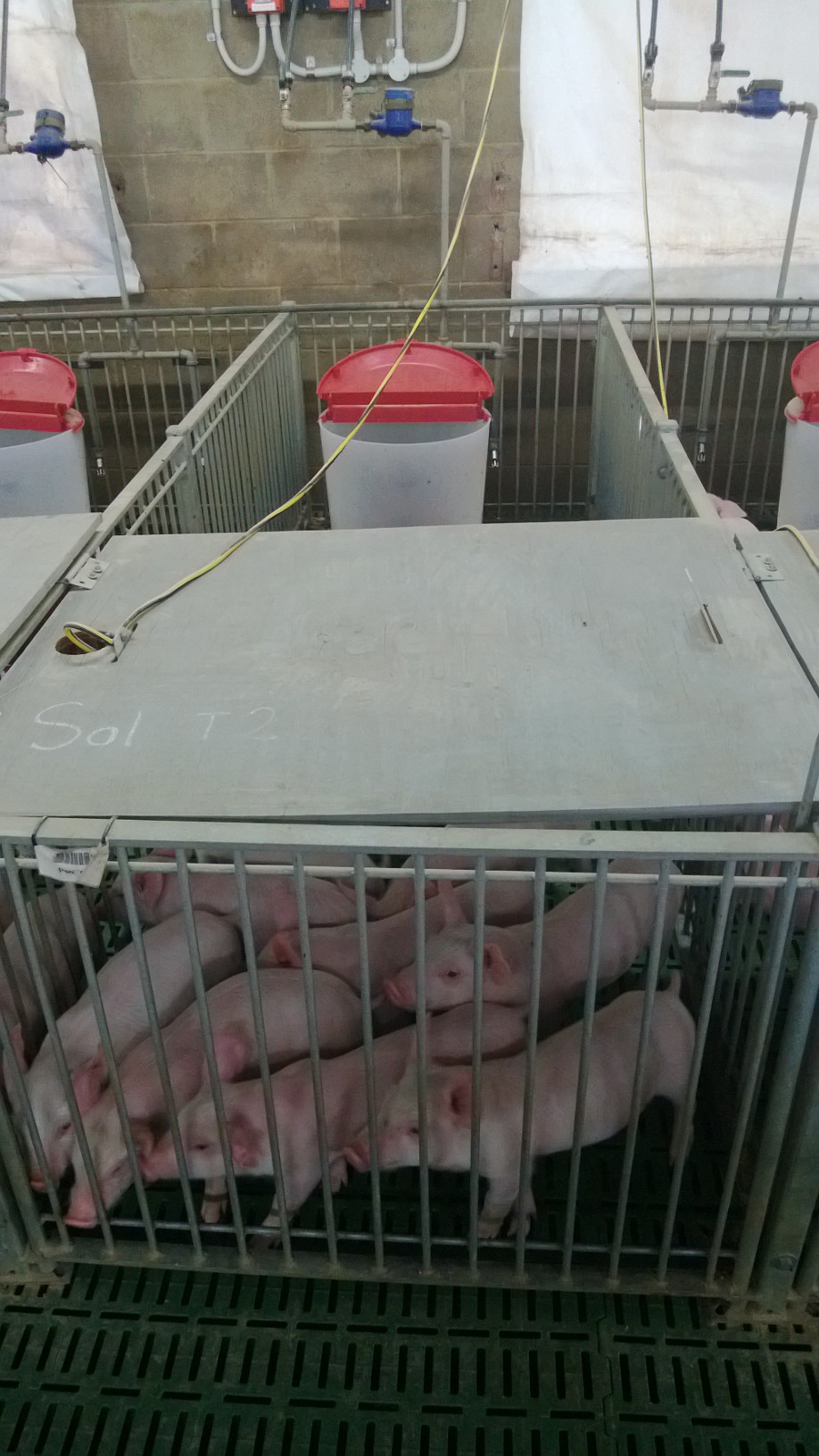

Supplement: Supplementary file 2 — Facility Pictures. (ZIP 2811 kb) [file 40813_2016_43_MOESM2_ESM.zip › Facility Pictures/Trial 4.jpg]

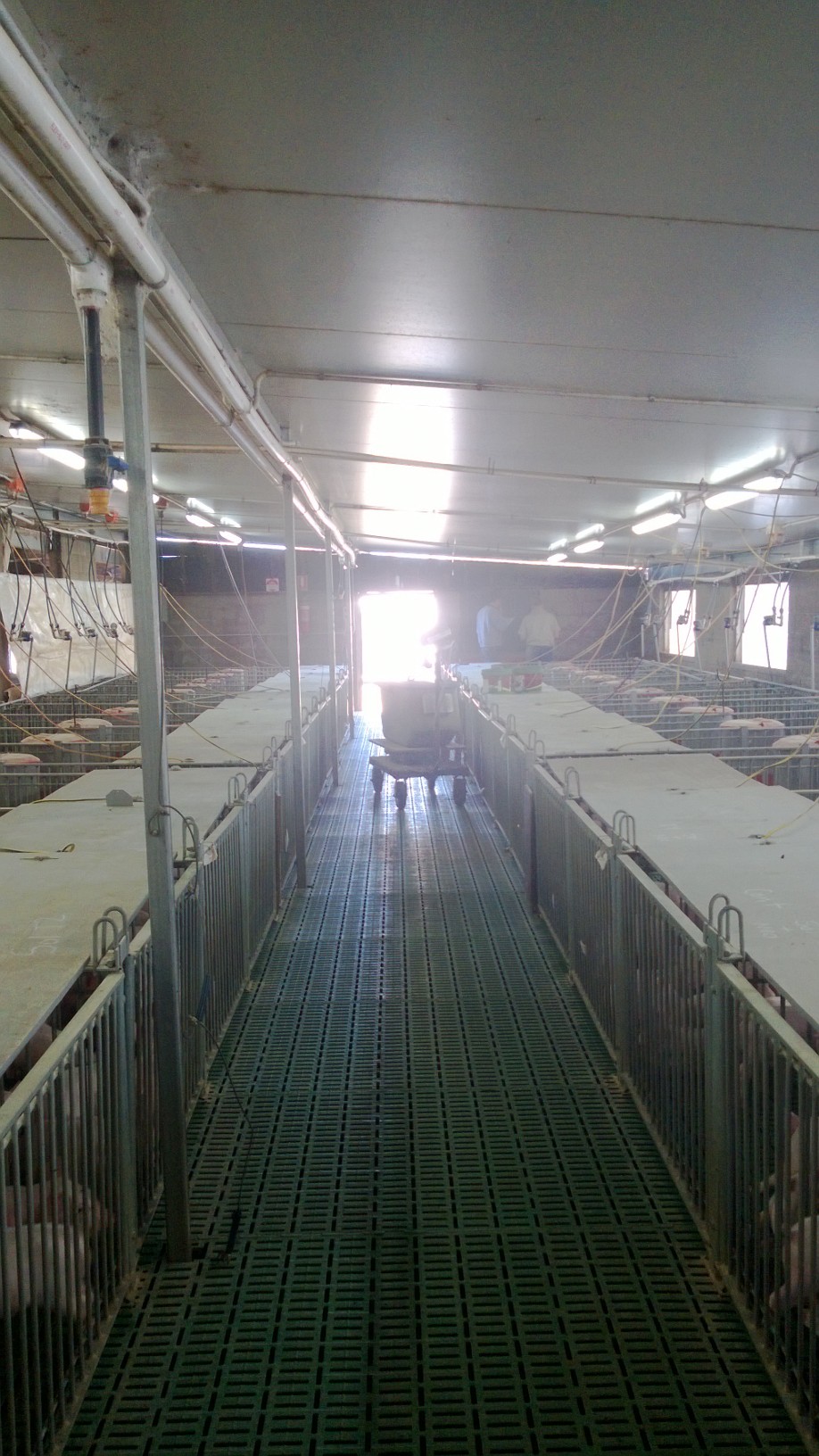

Supplement: Supplementary file 2 — Facility Pictures. (ZIP 2811 kb) [file 40813_2016_43_MOESM2_ESM.zip › Facility Pictures/Trial Shed.jpg]
